# Haploidentical Stem Cell Transplantation: A Gateway to Infrequent Availability of HLA-Matched Related Donors

**DOI:** 10.1155/2018/2573657

**Published:** 2018-09-10

**Authors:** Hafiz Muhammad Aslam, Shumaila Muhammad Iqbal, Hira Shaikh, Faizan A. Faizee, Ambreen A. Merchant, Marwan Shaheen, Shahrukh K. Hashmi

**Affiliations:** ^1^Department of Internal Medicine, Seton Hall University (St Francis) Program, Trenton, USA; ^2^Department of Internal Medicine, University at Buffalo (Catholic Health System-Sisters of Charity) Program, Buffalo, USA; ^3^Department of Internal Medicine, Allegheny Health Network, Pennsylvania, USA; ^4^Dow Medical College, Dow University of Health Sciences, Karachi, Pakistan; ^5^King Faisal University Hospital and Research Center, Riyadh, Saudi Arabia; ^6^Department of Medicine, Division of Hematology, Mayo Clinic, Rochester, MN 55905, USA

## Abstract

Haploidentical stem cell transplantation provides a plausible alternative for the patients when a fully matched donor is unavailable. Historically, the decision of considering haploidentical transplant has remained elusive; however, with the recent advances, the consideration of haploidentical grafts as a treatment option has become more apparent for both allografting for diseases and engraftment failure. We are reporting here an anecdotal case of a successful haploidentical engraftment in a patient with the prior graft failure of an HLA-matched related donor. Since the patient was severely alloimmunized, desensitization protocol was utilized before the haploidentical transplant, and the patient after 8 months of her second allogeneic transplantation is doing great with successful engraftment, no relapse, and no graft-versus-host disease (GVHD). Numerous reports pertinent to haploidentical graft have shown favorable outcomes in the graft placement, a decline in the rate of GVHD, and an improvement in the morbidity and mortality in affected individuals. Based on the current reports, haploidentical transplantation might be more feasible and has meaningful implications in the situations where matched donors are infrequent.

## 1. Introduction

Allogeneic hematopoietic cell transplantation (HCT) is a potentially curative treatment modality for hematological and nonhematological malignancies, primary immunodeficiency syndromes, and bone marrow failure syndromes. The pluripotent stem cells are acquired from the bone marrow or peripheral blood of an HLA-matched or HLA-unmatched donor in the cases of allogeneic HCT. Historically, matched-related donors were considered as the first preference for transplantation; however, only 20–30% patients have siblings that are HLA-identical [1, 2]. Matched-unrelated donors have been used with increasing frequencies in scenarios where matched-related donors are unavailable. *Excessive amount is consumed to coordinate with some registries internationally, though some registries have a quicker turn around. Nonetheless, the time required for a readily available family member is shorter than that required for finding an unrelated donor*; therefore, the chances of finding an unmatched donor for cases in which immediate HCT is required is lost [3]. HLA haploidentical stem cell transplantation (haplo HCT) is another alternative treatment modality where only one HLA haplotype is matched with an individual. The use of haplo HCT in the prior decade was mainly obscured by the high rates of graft failure, graft-versus-host disease, and infectious complications [4, 5]. Another predicament prohibiting its widespread use was the limited availability of prospective comparative studies. Furthermore, the presence of donor-specific antibodies (DSA) in the recipient created a significant hindrance to successful transplantation. Further clinical studies are ought to be done to contemplate the significance of haplo HCT and its preference over match-related and unrelated donors [6].

## 2. Case

A 40-year-old female, a known case of hypothyroidism, was discovered to have incidental pancytopenia while she was being managed for vocal cord papilloma at a tertiary care center. Extensive invasive workup for pancytopenia could not be ensued because of patient refusal. Almost a year later, the patient at 30 weeks of gestation presented to the hospital with severe fatigue. Her laboratory results revealed pancytopenia (white blood cell (WBC) count) of 2.8 × 10^9^/L, hemoglobin (Hb) of 9.5 g/dL, platelet (Plt) count of 150 × 10^9^/L, serum folate of 26 nmol/L, and serum B12 of 160 pg/mL along with normal renal/liver function profile and normal viral hepatitis serology reports. Peripheral blood morphology was performed, showing severe neutropenia and toxic granulation. Bone marrow biopsy demonstrated reduced cellularity (10–15%), reduced erythropoiesis, granulopoiesis, and megakaryocytes. Fluorescence *in situ* hybridization and flow cytometry studies revealed no evidence of bone marrow infiltration or leukemia.

Two months after uncomplicated normal vaginal delivery, the patient presented to the hospital with aplastic anemia, evident by her complete blood count: WBC of 1 × 10^9^/L, absolute neutrophil count (ANC) of 0.05, Hb of 7.8 g/dL, and Plt of 5 × 10^9^, and the bone marrow biopsy revealed markedly reduced cellularity with many lymphoid aggregates and no clear dysplastic changes or evidence of malignancy. Molecular study (BCR-ABL by PCR), cytogenetic study (Karyotyping), and chromosome breakage study were performed. The molecular study and chromosome breakage study came negative, and there was no growth on the karyotyping by G-banding.

The patient was kept under observation in the hospital while tests were performed on her family to search for a HLA-matched donor. Her brother was subsequently found to be a full HLA-match. The preparative regimen for transplantation consisted of cyclophosphamide (total dose: 200 mg/kg) and along with antithymocyte globulin (ATG; thymoglobulin Genzyme^©^).

The patient developed a fever on the 10th day of transplantation with skin redness and swelling on the intravenous line sites. She was administered cefepime and vancomycin, which was later switched to meropenem (blood cultures revealed positive *Pseudomonas aeruginosa* colonies). Furthermore, ultrasonography was performed, showing edema with mildly increased vascularity in accordance with inflammation and no signs of abscess. Skin biopsy was done, indicating dermal edema and dermal RBC extravasation. She also developed cyclosporine-related hypertension which was managed by amlodipine administration. The patient was started on granulocyte colony stimulating factor (G-CSF) at 300 mcg daily.The patient's cell counts did not rise significantly (WBC of 0.18 × 10^9^/L, Hb of 9.8 mg/dL, and Plt of 2 × 10^9^/L) ten days after administration of G-CSF; therefore, the dosage of G-CSF was further increased to 5 mcg/kg/day. Chimerism studies at day +30 after transplantation indicated *25% donor lymphoid cell engraftment on peripheral blood*, and bone marrow (BM) biopsy showed a cellularity of 5%. The patient was eventually declared as graft failure.

The patient's state of health was discussed in the BMT didactic conference; the team decided to consider the patient for salvage haploidentical HCT after conditioning. *It is worthwhile in general to wait until day +60 for engraftment failure before taking a major step like a second allogeneic transplant*; *however, the patient's risk of infections was significantly high, and thus waiting for an extra month would have possibly led to fatal infections. Thus, workup for second allogeneic transplant started around 5 weeks from the first transplant*. The conditioning regimen comprised of antithymocyte globulin (ATG), total body irradiation (TBI), and fludarabine. *ATG brand THYMOGLOBULIN (Genzyme) at a dose of 9 mg was used in conditioning. Same brand was used for both transplants. DSAs before the second transplant to the selected donor were found in DRB1 and DQB1 as follows: anti-DRB1 mean fluorescence intensity (MFI) = 3154 and anti-DQB1 MFI = 2141*.

The patient received haploidentical stem cells from her other sibling. She received CD34+ count of 11.12 × 10^6^/kg after conditioning with the aforementioned conditioning regimen. *Total time difference between the 2 transplants was 65 days (first allogeneic transplant: 20th December 2016; second allogeneic transplant: 23rd February 2017)*. After transplantation, the patient remained asymptomatic with no signs or symptoms suggestive of acute GVHD or infections in acute posttransplant phase. Her laboratory results demonstrated ANC > 0.5 and platelet >200 × 10^9^/L at day +10 after transplantation. The patient was further managed with intensified immunosuppressive therapy, which includes tacrolimus and mycophenolate. The patient was discharged on pertinent medications and was advised to follow up with her primary care physician.

Five months after transplantation, the patient presented to the hospital with fever and diarrhea for 3 days; it was initially managed with antibiotics. However, her condition deteriorated without significant improvement. On performing further investigations, she was found to have *Clostridium difficile* (*C. diff*) positive stool, asymptomatic urinary tract infection (UTI) evident by *E. coli* positivity in urine culture report, acute kidney injury (AKI) possibly due to dehydration, and elevated tacrolimus blood levels. The patient's condition gradually improved after administration on antibiotics (metronidazole and ciprofloxacin for C. diff and UTI, respectively) and was eventually discharged.

In order to rule out GVHD, colonoscopy was performed; it demonstrated moderate ulcerative colitis, which came out negative for mucosal architectural distortion and ulceration, microorganism viral cytopathic effect, granuloma, and adenomatous dysplastic change. Typical morphological features of acute or chronic GVHD were not evident on colonic biopsy; a microbiological correlation was suggested to rule out the possibility of infectious colitis. Her laboratory results revealed the following: white blood cell (WBC) of 5.9 × 10^9^/L, hemoglobin (Hb) of 13.2 mg/dL, platelets (Plt) of 189 × 10^9^/L, and absolute neutrophil count (ANC) was normal. Renal function displayed improvement showing a precipitous decline in blood creatinine levels from 300 *µ*mol/L to 74 *µ*mol/L, GFR turned out to be greater than 60 mL/min/1.73 m^2^, and potassium levels were 4.4 mm/L. Tacrolimus levels were found to be therapeutic (serum level: 10.9 ng/mL).

Her dose of tacrolimus was reduced, keeping in consideration the blood levels of tacrolimus. The patient was subsequently discharged on pertinent medications and was instructed to follow up with her primary care physician.

## 3. Discussion

Matched-related and matched-unrelated donor sources have served as a gold standard for many years in the field of HCT. The outcome of HCT has been proven to be greatly affected by the HLA incompatibilities between the host and the donor; the problem is dealt by finding a donor that is HLA compatible with the recipient. Due to the limited availability of HLA-matched donor, and the time spent to find matched-unrelated donors, haploidentical donors have provided us with a reasonable alternative because almost everyone has at least one haploidentical relative. Haploidentical transplantation method is considered to be a relatively novel technique but it poses challenges due to the limited availability of the clinical data.

Several studies using haploidentical grafts resulted in high rates of graft failure in the past, leading to a decline in the use of haploidentical grafts. With recent advances, several strategies are implicated like administration of posttransplant immunosuppressive agents, and reduced intensity conditioning regimens have yielded encouraging results in the survival of patient receiving haploidentical grafts [5, 7–9].

Based on a study by Esteves et al., transplant-associated fatalities due to bacterial sepsis, pulmonary aspergillosis, viral infections, toxoplasmosis, and severe GVHD can negatively influence a patient's prognosis with haplo HCT. The risk of severe GVHD occurrence can be abrogated by posttransplant cyclophosphamide to quite an extent [10]. Studies on HCT in severe refractory aplastic anemia, when posttransplant cyclophosphamide was utilized, have revealed remarkably less morbidities like GVHD, which are generally sensitive to steroids [5]. Another study demonstrated that the patients with secondary graft failure, at the time of neutrophil engraftment, received salvage transplantation which caused complete donor chimerism. *Overall, 15 patients underwent neutrophilic engraftment out of which two died and one of the patient started on ELTROMBOPAG, after which cell count starts to improve. Remaining 12 obtained the platelet engraftment*. Amidst 12, two went for salvage haplo HCT from a different donor due to secondary failure. Only one patient experienced a grade 4 GVHD and demanded treatment for refractory GVHD. Each patient died of transplant related mortality with no relapse. Those with hematological malignancies have a higher incidence of relapse following haplo HCT [10]. Another study showed 30% of patients with hematological malignancies like leukemia and lymphoma exhibited grade 3–5 toxicities after haplo HCT. Median follow-up after transplant was 375 days, and relapse was the cause of death [11]. Most prevalent infection after the administration of pretransplant cyclosporine was the reactivation of CMV. *Majority of the patients died due to transplant-related mortality such as GVHD, regimen-associated toxicity, primitive graft failure, and EBV, respectively* [12]. At approximately a year of enduring myeloid engraftment, 89 patients of haploidentical donor revealed increase combined incidence of 2–4 aGVHD, 3–4 aGVHD, and chronic GVHD but similar rate of chronic extensive GVHD. Increased amount of transfusion and lower performance status before HCT unfavorably influenced the overall survival rate [12].

Based on the evidence-based data, we initially grafted a matched-related specimen in our patient to ensure the least chances of graft rejection; nevertheless, the failure led us to haploidentical grafting, and it exhibited surprisingly affirmative result. Engraftment was stable without any clinical evidence of GVHD. Razari et al. reported successful haploidentical engraftment in approximately 94% of patients with hematological malignancies. For this series, they selected adult patients with nonmyeloablative transplant using 3/6 HLA-matched family donors [13]. The series demonstrated promising result in the survival of the patient in comparison with matched-related/unrelated donor. O'Donnell et al. conducted unrelated-haploidentical transplant with immunosuppression with cyclophosphamide which showed favorable results in promoting engraftment and preventing GVHD [14].

Haplo HCT could be a good alternative in transplantation, as several case reviews have shown encouraging outcomes and improved survival rates amongst the patients. Further clinical studies are warranted to reach a more definite conclusion. Our case represents favorable results that haploidentical engraftment might have on patients' survival after engraftment failure from the first allograft. It is, however, too early to draw definite conclusions to consider this as a standard of care for engraftment failure. Further advances and more prospective clinical studies might turn out in the favor of haploidentical engraftment procedures. Additionally, many different desensitization strategies are being utilized by different institutions globally [15].

## 4. Conclusion

Numerous studies pertinent to haploidentical graft have shown favorable outcomes in the graft placement, a decline in the rate of GVHD, and an improvement in the morbidity and mortality in affected individuals. Based on the current reports, haploidentical HCT might be more feasible and has meaningful implications in the situations where matched donors are infrequent for engraftment failure.
